# Direct Printing of Ultrathin Block Copolymer Film with Nano‐in‐Micro Pattern Structures

**DOI:** 10.1002/advs.202303412

**Published:** 2023-08-21

**Authors:** Tae Wan Park, Young Lim Kang, Eun Bin Kang, Hyunsung Jung, Seoung‐Ki Lee, Geon‐Tae Hwang, Jung Woo Lee, Si‐Young Choi, Sahn Nahm, Se‐Hun Kwon, Kwang Ho kim, Woon Ik Park

**Affiliations:** ^1^ Department of Materials Science and Engineering Korea University Seoul 02841 Republic of Korea; ^2^ Department of Materials Science and Engineering Pukyong National University (PKNU) 45 Yongso‐ro, Nam‐gu Busan 48513 Republic of Korea; ^3^ Nano Convergence Materials Center Korea Institute of Ceramic Engineering & Technology (KICET) Jinju 52851 Republic of Korea; ^4^ School of Materials Science and Engineering Pusan National University (PNU) Busan 46241 Republic of Korea; ^5^ Department of Materials Science and Engineering Pohang University of Science and Technology (POSTECH) Pohang 37673 Republic of Korea; ^6^ Global Frontier R&D Center for Hybrid Interface Materials (HIM) Pusan National University Busan 46241 Republic of Korea

**Keywords:** block copolymer, nanopatterning, nanotransfer printing, self‐assembly, wafer scale

## Abstract

Nanotransfer printing (nTP) is one of the most promising nanopatterning methods given that it can be used to produce nano‐to‐micro patterns effectively with functionalities for electronic device applications. However, the nTP process is hindered by several critical obstacles, such as sub‐20 nm mold technology, reliable large‐area replication, and uniform transfer‐printing of functional materials. Here, for the first time, a dual nanopatterning process is demonstrated that creates periodic sub‐20 nm structures on the eight‐inch wafer by the transfer‐printing of patterned ultra‐thin (<50 nm) block copolymer (BCP) film onto desired substrates. This study shows how to transfer self‐assembled BCP patterns from the Si mold onto rigid and/or flexible substrates through a nanopatterning method of thermally assisted nTP (T‐nTP) and directed self‐assembly (DSA) of Si‐containing BCPs. In particular, the successful microscale patternization of well‐ordered sub‐20 nm SiO*_x_* patterns is systematically presented by controlling the self‐assembly conditions of BCP and printing temperature. In addition, various complex pattern geometries of nano‐in‐micro structures are displayed over a large patterning area by T‐nTP, such as angular line, wave line, ring, dot‐in‐hole, and dot‐in‐honeycomb structures. This advanced BCP‐replicated nanopatterning technology is expected to be widely applicable to nanofabrication of nano‐to‐micro electronic devices with complex circuits.

## Graphical abstract

A dual nanopatterning process of block copolymer self‐assembly and transfer‐printing can create periodic sub‐20 nm structures on an eight‐inch wafer scale by the printing of patterned ultrathin block copolymer film onto desired substrates. Various complex geometries of nano‐in‐micro structures, specifically angular/wave lines, rings, and dot‐in‐hole/dot‐in‐honeycomb patterns consisting of sub‐20 nm SiO*_x_* structures are successfully generated by the combined patterning method.

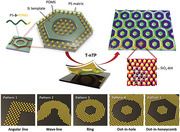

## 1 Introduction

The development of a nanopatterning process with a high pattern resolution, a low‐cost, and high throughput is essential for the nanofabrication of various electronic devices with integrated circuits.^[^ ^1^, ^2^, ^3^, ^4^, ^5^ ^]^ Currently, photolithography and optical lithography are the nanopatterning methods most widely used to create various geometric nano‐to‐micro patterns with designed photomasks with complex circuits.^[^ ^6^, ^7^, ^8^ ^]^ Photolithography allows most materials to be reliably patterned with the use of light‐sensitive chemical photoresists, providing precise control over the pattern shape and size.^[^ ^9^, ^10^, ^11^ ^]^ However, this method has several critical disadvantages related to, for instance, the physical resolution, the high cost and expensive equipment, and a lack of good patternability for non‐planar surfaces.^[^ ^10^ ^]^ To resolve these issues, for the last several decades various alternative nanopatterning technologies, such as extreme ultraviolet lithography,^[^ ^12^, ^13^, ^14^ ^]^ atomic force microscope lithography,^[^ ^15^, ^16^ ^]^ dip‐pen lithography,^[^ ^17^, ^18^ ^]^ directed self‐assembly (DSA),^[^ ^19^, ^20^, ^21^, ^22^ ^]^ nanoimprint lithography,^[^ ^23^, ^24^, ^25^, ^26^ ^]^ and nanotransfer printing (nTP)^[^ ^27^, ^28^, ^29^, ^30^ ^]^ have been consistently developed to obtain designed nanopatterns effectively with high precision levels for smart device nanofabrication. Among these lithographic methods, the nTP process shows excellent throughput patternability with high pattern resolutions, effectively generating various nanostructures on both planar and non‐planar surfaces at a low process cost using a rigid mold with nano‐to‐microscale patterns.^[^ ^31^, ^32^, ^33^ ^]^ Hence, many patterning research groups have suggested several innovative and useful nTP methods to produce a variety of 2D and 3D pattern geometries.^[^ ^34^, ^35^, ^36^ ^]^ However, the advantages of these nTP methods, they still face the important challenge of the pattern resolution limit, which strongly depends on the pattern size of the master mold.

As one of mold strategies that can be used to realize a high‐density pattern, DSA of Si‐containing block copolymers (BCPs) with a high Flory–Huggins segmental interaction parameter (*χ*) can provide the useful fabrication of rigid molds with ultra‐small nanopatterns.^[^ ^37^ ^]^ Over the past several decades, the DSA of BCPs has been studied extensively as a promising next‐generation nanopatterning method due to its excellent resolution, pattern scalability, cost‐effective process, and numerous pattern geometries.^[^ ^38^ ^–45]^ The self‐assembly of BCPs, which consist of two or more immiscible polymer blocks, can create various nanostructures with ultra‐fine feature sizes of 5–50 nm, such as spheres, cylinders, and hexagonally perforated lamella (HPL), through a spontaneous microphase separation process.^[46–50]^ In particular, self‐assembled Si‐containing poly(styrene‐*b*‐dimethylsiloxane) (PS‐*b*‐PDMS) BCPs with high‐*χ* values can be easily converted into rigid sub‐20 nm SiO*_x_* nanostructures after a short O_2_ plasma treatment.^[51–54]^ As previously reported by the authors, using a self‐assembled SiO*_x_* line structure as the master mold, we fabricated a high‐density cross‐bar NiO*_x_*/Pt resistive memory device on a flexible substrate that demonstrated unipolar resistive switching behavior through a thermally assisted nTP (T‐nTP) process.^[55]^ We also demonstrated the large‐area patterning of various nanostructures on the eight‐inch scale via T‐nTP using a rolling press system to provide uniform pressure and heat. After the replication of various patterns from an eight‐inch Si wafer, functional materials on a polymer replica pattern were successfully transfer‐printed onto large target substrates through uniform heat injection and pressure steps.

However, despite the advantages of these nTP methods, they basically have a significant drawback in that they mostly require the use of a replication processing step. Also, it is necessary to form an active layer on the replicated pattern through a physical vapor deposition (PVD) process.^[55,56]^ This implies that the materials replicated from molds with fine patterns cannot be used directly as functional structures for device applications. For these reasons, the development of replica materials with good functionalities should be encouraged to extend the versatility of the nTP process to the smart fabrication of nanodevices without using a BCP structure as a master mold. Particularly, an advanced dual patterning strategy consisting of the nTP and DSA processes using various BCP thin films as replica materials can realize direct pattern generation with both good functionalities and ultra‐fine resolutions. Accordingly, a PVD‐free nTP method capable of nanopatterning should be developed using ultra‐thin self‐assembled BCP replication patterns for the successful implementation of functional devices.

Here, we demonstrate a useful and reliable dual nanopatterning method capable of generating well‐ordered sub‐20 nm structures with good functionalities on the eight‐inch wafer scale by the transfer‐printing of ultra‐thin BCP patterns onto rigid and flexible substrates. In particular, we show how to transfer templated PS‐*b*‐PDMS BCPs nanostructures from a guiding Si mold onto desired surfaces by controlling the film thickness of BCPs, the annealing conditions, and the printing temperature, all of which cannot be done when using conventional nanopatterning methods. We also exhibit a variety of complex pattern geometries of nano‐in‐micro structures through the well‐defined microscale patternization process by the T‐nTP of BCPs, showing angular line, wave line, ring, dot‐in‐hole, and dot‐in‐honeycomb patterns consisting of self‐assembled sub‐20 nm SiO*_x_* nanoparticles. In addition, we demonstrate reliable pattern formation on the eight‐inch wafer scale for transfer‐printed sub‐micron patterns composed of highly ordered sub‐10 nm line structures.

## 2 Results and Discussion

**Figure 1:**
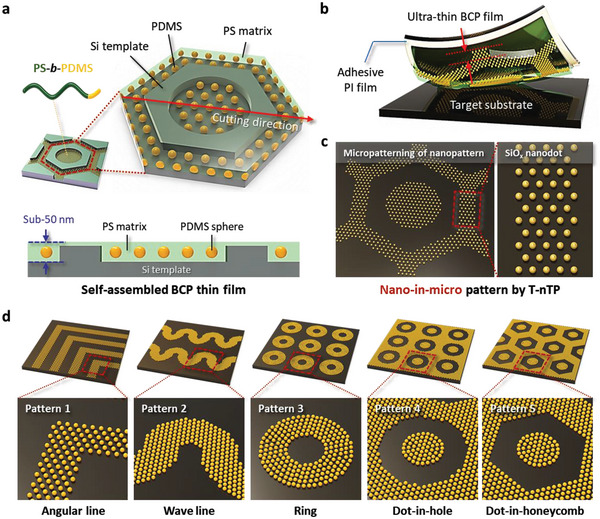
Microscale patternization of self‐assembled PS‐*b*‐PDMS BCP thin film. a) Self‐assembled PS‐*b*‐PDMS BCP thin film with a sub‐50 nm thickness directed by a Si guiding template. b) Transfer‐printing of replicated ultra‐thin BCP film onto a target substrate. c) Microscale patternization of self‐assembled SiO*_x_* dot nanostructure by T‐nTP, showing a nano‐in‐micro pattern. d) Various microscale patterns consisting of self‐assembled SiO*_x_* dots, showing angular line, wave line, ring, dot‐in‐hole, and dot‐in‐honeycomb nano‐in‐micro patterns.

**Figure** **1** shows the microscale patternization of a self‐assembled PS‐*b*‐PDMS BCP structure realized by the T‐nTP process. The procedure of the T‐nTP method to create periodic complex patterns consisting of highly ordered SiO*_x_* nanospheres is schematically shown in Figure S1 (Supporting Information). It consists of two sequential steps of the DSA of BCP (step I) and the transfer‐printing of the BCP pattern (step II). In step I, ultra‐thin BCP film is formed on a topographically patterned guiding template (Si) fabricated by conventional photolithography using a spin‐coater. Then, the BCP thin film is solvent‐annealed at a warm temperature (≈65 °C) to obtain high‐resolution BCP dot structures within the designated area of the Si template (see Experimental Method for the details of the DSA process). In step II, microscale‐patterned BCP thin film is produced by peeling off the BCP film from the Si master mold using an adhesive polyimide (PI) film. The BCP film is transfer‐printed onto the target substrate by the T‐nTP method and subsequently dry‐etched with CF_4_ and O_2_ plasma, showing a well‐defined nano‐in‐micro SiO*_x_* (oxidized PDMS) pattern.

To obtain sub‐20 nm nanostructures, we chose a sphere‐forming PS‐*b*‐PDMS BCP with a high‐*χ* value as a functional replica material. As previously reported by the authors, the self‐assembled SiO*_x_* nanostructures which are thermally stable show resistive switching behavior and therefore they can be used as active materials for non‐volatile memory device applications.^[57]^ To obtain a mono‐layered SiO*_x_* nanostructure, the thickness of BCP film on the patterned Si template (or mold) is very important. Figure 1a shows the self‐assembled PS‐*b*‐PDMS BCP mono‐layer, indicating the confinement of hexagonally ordered PDMS spheres in the PS matrix. In general, ultra‐thin BCP film with a thickness of ≈40 nm can be self‐assembled into a mono‐layered periodic SiO*_x_* nanopattern through microphase separation and a dry‐etching process. The BCP patterns replicated from the Si mold by adhesive PI film can be reliably transfer‐printed onto rigid and/or flexible surfaces by the T‐nTP process, resulting in the formation of microscale patterns consisting of SiO*_x_* nanostructures (Figure 1b,c). In this study, we refer to this process as dual nanopatterning using T‐nTP and the DSA of BCP. The pattern geometry of the BCP film can be controlled by managing the pattern shape of the Si template, while the BCP morphology depends on the volume fraction of the BCP. As shown in Figure 1d, various nano‐in‐micro patterns can be generated by T‐nTP using Si templates with diverse patterns, such as angular lines, wave lines, rings, dot‐in‐hole patterns, and dot‐in‐honeycomb patterns. Here, it should be noted that this approach allows various patterns of self‐assembled BCP nanostructures to be formed on planar and non‐planar substrates without any guiding structure.

**Figure 2:**
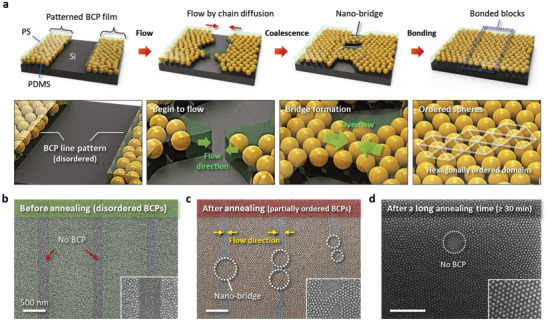
Microphase separation of the patterned BCP thin film by T‐nTP. a) Time‐evolution of a printed SD56 BCP line pattern during solvent annealing, showing the formation process of a BCP nano‐bridge by chain diffusion. b) Micro‐patterned BCP thin film after transfer‐printing onto the Si substrate by T‐nTP, showing disordered PDMS microdomains before the annealing process. c) Nano‐bridged BCP line pattern through the BCP flow into BCP‐empty spaces, showing locally ordered PDMS microdomains after annealing. The printed BCP lines begin to bridge the gaps or spaces by means of solvent‐induced chain diffusion. d) Coalescence of patterned‐BCP thin film after a long annealing time (≥ 30 min), showing connected BCP microdomains.

**Figure** **2** and Figure S2 (Supporting Information) show a discrete ultra‐thin BCP line pattern using a line‐shaped Si template with a depth of 40 nm created by the replication step of the T‐nTP method. To obtain a sub‐20 nm SiO*_x_* nanodot pattern, we employed a sphere‐forming PS‐*b*‐PDMS BCP (SD56, *χ* = ≈0.26) with a molecular weight (MW) of 56 kg mol^−1^ and with a relatively low PDMS volume fraction (*f*_PDMS_) of 16%. We used a thermally assisted solvent vapor annealing process to efficiently expedite the slow self‐assembly of high‐χ BCPs through rapid diffusion of solvent molecules into BCP film. The patterned BCP line structure was solvent‐annealed at 65 °C by toluene vapor to produce self‐assembled PDMS spheres. During the annealing process, the ordering of the self‐assembled PDMS microdomains was increased, whereas the patterned BCP line structure was connected by the flow of the BCP, resulting in the unwanted formation of nanoscale bridges, as shown in Figure 2a. This means that in order to obtain a well‐ordered BCP nanostructure, a templated annealing process must be performed before transfer‐printing the BCP film.^[58,59]^ Figure 2b presents the transfer‐printed BCP line/space patterns (line‐width: 1 µm, space‐width: 250 nm) before the annealing process, showing the poorly ordered SiO*_x_* dot structure. After annealing process at 65°C for 10 min, the patterned BCP structure begins to flow into the space region without a BCP microdomain, leading to the collapse of the locally ordered BCP line pattern, as shown in Figure 2c. This stems from the movement of the BCP line structure into the empty space, as encouraged by solvent‐induced chain diffusion during the self‐assembly process of the BCP, although the ordering of the self‐assembled BCP is increased. After annealing for a long annealing time of 30 min, total coalescence of the patterned‐BCP lines was observed, resulting in a connected BCP thin film consisting of semi‐ordered PDMS spheres, as shown in Figure 2d. Figure S3 (Supporting Information) shows a self‐assembled BCP structure on the planar surface, which is similar to the fully collapsed BCP line structures due to the absence of guiding template. Here, we find that the annealing process should be performed before the T‐nTP process to obtain well‐defined nano‐in‐micro structures.

**Figure 3:**
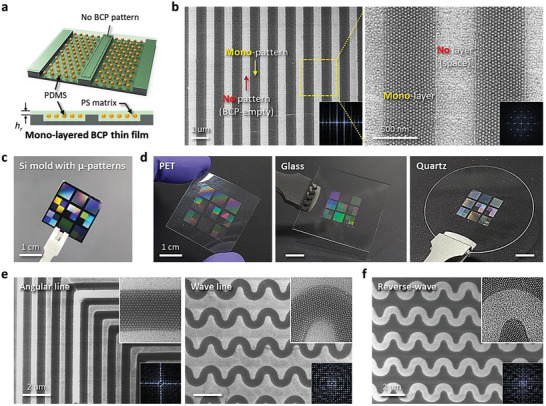
Formation of various microscale patterns consisting of periodic sub‐20 nm SiO*_x_* nanostructures on the transparent substrates by T‐nTP. a) Schematic image of self‐assembled ultra‐thin BCP film after solvent annealing. b) Discrete microscale SD56 BCP line pattern consisting of self‐assembled PDMS spheres in a PS matrix, showing a highly ordered SiO*_x_* dot pattern after O_2_ plasma treatment. The inset images show fast Fourier transform (FFT) patterns of the corresponding SEM images, indicating the well‐ordered microscale SD56 BCP line consisting of SiO*_x_* nanodots. c) Chip‐scale Si mold with various microscale patterns. d) Photo images of various transfer‐printed SD56 BCP patterns on transparent substrates of PET, glass, and quartz using a Si mold with BCP micro‐patterns realized by T‐nTP. e,f) SEM images of ultra‐thin SD56 BCP patterns on PET surfaces. The magnified SEM images and FFT patterns show the hexagonally ordered SiO*_x_* nanoparticles in the transfer‐printed microscale angular line, wave line, and reverse‐wave patterns.

**Figure** **3**a shows a schematic image of the self‐assembled BCP mono‐layer film after solvent annealing. Prior to the transfer‐printing of BCP thin film, we obtained periodic SD56 BCP by controlling the annealing conditions of time and temperature at toluene vapor. The dual pattern generation of a well‐defined SD56 line pattern with discrete lines (width of 500 nm) composed of hexagonally ordered sub‐20 nm SiO*_x_* dot structure can be realized by transfer‐printing and subsequently dry etching after an annealing process under the optimum conditions (at 65 °C for 10 min), as shown in Figure 3b. The inset images of fast Fourier transform (FFT) patterns clearly show the excellent ordering of microscale SD56 BCP lines along with SiO*_x_* dot patterns within the microscale BCP lines. The reason why the discrete BCP lines can be formed is that the residual thickness (*h*_r_) of the SD56 BCP is insufficient to cause microphase separation of the BCP. A pattern with multiple BCP lines can also be obtained by controlling the thickness of the SD56 BCP film. For the pattern formation of multi‐layered BCP structure, a longer annealing time of 30 min was required to increase polymer chain mobility or chain diffusivity than mono‐layered BCP. The formation of an interconnecting line pattern consisting of mono‐layer and double‐layer SiO*_x_* dot structures was achieved using an SD56 BCP with a higher weight percent (2 wt.%), as shown in Figure S4 (Supporting Information). The pattern geometry of BCP thin film depends on the shape of the pattern engraved on the Si master mold. When using the SD56 BCP solution with a higher weight percent, the transfer printing result shows an interconnected BCP film rather than individual BCP lines (thickness of transfer‐printed BCP film: ≈80 nm, see Figure S5, Supporting Information). Figure 3c shows a chip‐scale Si mold with various microscale patterns with a depth of 40 nm. We can realize the diversification of patterned BCP thin film by using a Si mold with micro‐patterns. Figure 3d shows a patterned BCP thin film consisting of SiO*_x_* nanodots on transparent polyethylene terephthalate (PET), slippery glass, and quartz surfaces using a Si mold with micro‐patterns created by the T‐nTP process, which was obtained by transfer‐printing of the patterned replica BCP film (Figure S6, Supporting Information). Figure S7 (Supporting Information) displays the effects of the temperature dependency on the transfer yield of a replicated ultra‐thin BCP film, demonstrating perfect patterning without a non‐patterned area at 120 °C (threshold temperature, *T*_TH_). If temperatures over *T*_TH_ (> 120 °C) are injected during the T‐nTP process, the transfer‐printed patterns collapse and are crushed despite the successful printing of the BCP film. Here, it should be strongly emphasized that functional ultra‐thin BCP patterns can be replicated and transfer‐printed on both rigid and flexible surfaces without using nanomolds with sub‐20 nm feature sizes. Figure 3e,f show well‐defined nano‐in‐micro patterns of angular, wave, and reverse‐wave structures consisting of SiO*_x_* nanodots on a PET surface. The magnified scanning electron microscope (SEM) images and FFT patterns indicate the highly ordered SiO*_x_* nanoparticles in the transfer‐printed microscale angular line, wave line, and reverse‐wave patterns.

**Figure 4:**
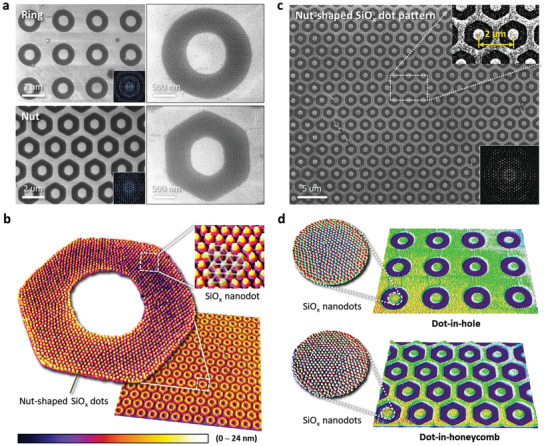
Various pattern formation of complex nano‐in‐micro structures by T‐nTP of self‐assembled BCP. a) (Left, upper) Ring and (Left, lower) nut‐shaped sub‐20 nm SiO*_x_* nanodots. (Right) Magnified images of ring/nut‐shaped SiO*_x_* nano‐in‐micro structures. The SEM images show well‐defined ring and nut microscale patterns consisting of self‐assembled SiO*_x_* nanodot structures. b) Surface plotted 3D images of a nut‐shaped SiO*_x_* nanodot structure. The image clearly presents discrete nut‐shaped SiO*_x_* nanoparticles, showing a hexagonally arranged SiO*_x_* dot pattern. c) Large‐area patterning of the nut‐shaped SiO*_x_* structure. The inset images show FFT patterns of the corresponding SEM images, presenting well‐ordered microscale ring and nut patterns. d) Complex dot‐in‐hole and dot‐in‐honeycomb patterns consisting of SiO*_x_* nanodots. Both patterns show the reverse shapes of their respective ring and nut patterns.

**Figure** **4** shows the formation of various patterns of complex nano‐in‐micro structures by the T‐nTP of self‐assembled BCP. Shown in the figure are ring, nut, dot‐in‐hole, and dot‐in‐honeycomb structures. Figure 4a shows well‐defined ring and nut‐shaped patterns with a width of 500 nm consisting of sub‐20 nm SiO*_x_* nanodots created through the micro‐patterning process of self‐assembled SD 56 BCP thin film. 3D surface plot images clearly show hexagonally ordered SiO*_x_* nanoparticles in an individual nut‐shaped hexagonal pattern array, as shown in Figure 4b. Figure 4c shows the large‐area patterning result of a nut‐shaped SiO*_x_* nanostructure created by the dual patterning method. Figure S8 (Supporting Information) presents the size distribution of center‐to‐center (C‐to‐C, period or pitch) of transfer‐printed nut‐shaped SiO*_x_* nanodots. Complex pattern generation of the reverse shapes of rings and nuts can also be achieved through the same process by using inverse‐patterned Si molds (Figure S9, Supporting Information). Figure 4d shows the well‐defined 3D morphologies of complex dot‐in‐hole and dot‐in‐honeycomb patterns composed of SiO*_x_* nanodots. These transfer‐printed unusual pattern structures show excellent pattern uniformity (Figure S10, Supporting Information). Here, it should also be noted that various, complex dual patterns of a sub‐20 nm nanostructure can be effectively created by the combined patterning process of T‐nTP and BCP self‐assembly within individual microscale patterns. Such patterns cannot be obtained by general lithography processes.

**Figure 5:**
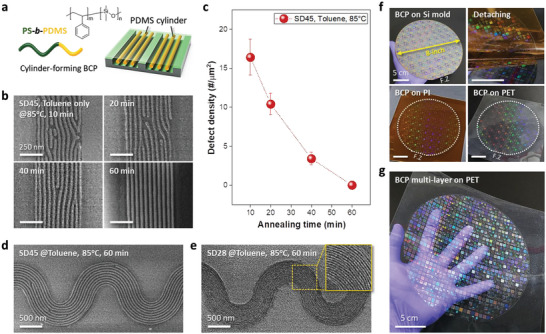
Pattern formation of self‐assembled sub‐20 nm SiO*_x_* line structures in microscale patterns via the T‐nTP process on the eight‐inch wafer scale. a) Schematic image of a self‐assembled line structure using a cylinder‐forming PS‐*b*‐PDMS BCP. b) Self‐assembled sub‐20 nm SiO*_x_* line structures in a patterned SD45 BCP line at different annealing times, depending on the time before the transfer‐printing process. c) Manually counted defect density of self‐assembled SD45 SiO*_x_* line structures annealed at varied annealing times. d) Wave‐shaped SD45 BCP pattern consisting of a highly ordered SiOx line structure annealed for 60 min. e) Wave‐shaped SD28 BCP pattern consisting of a self‐assembled sub‐10 nm SiOx line structure annealed for 60 min using a cylinder‐forming SD28 BCP. f) Process sequence for the microscale patternization of ultra‐thin BCP nanopatterns on an eight‐inch wafer. The self‐assembled BCP thin film on the eight‐inch Si mold was transfer‐printed onto a PET surface by the T‐nTP process using a laminating system. (Right, lower) This photo image obviously shows a well‐defined BCP thin film on PET on the eight‐inch scale realized by the T‐nTP method. g) Transfer‐printed multi‐layered BCP film on PET via T‐nTP using a patterned Si mold with a greater depth (≈ 250 nm).” on page 20 of the revised manuscript.

To extend the versatility of the morphological nanostructures in the micro‐patterns, we used cylinder‐forming PS‐*b*‐PDMS BCPs (SD45 and SD28) with an MW of 45 and 28 kg mol^−1^ (*f*_PDMS_ = 33.7% and 32.1%), respectively. **Figure** **5** shows the pattern formation process of self‐assembled SiO*_x_* line structures with a sub‐20 nm line‐width in microscale patterns on the eight‐inch wafer scale created by the T‐nTP process. Figure S11 (Supporting Information) shows a disordered SiO*_x_* line structure with a line width of sub‐20 nm on a planar surface without a guiding template. Figure 5a depicts self‐assembled PDMS lines realized using a cylinder‐forming BCP within a surface‐patterned guiding template. Figure 5b shows the morphology evolution of self‐assembled SiO*_x_* line structure with a 16 nm line width in a micro‐patterned SD45 BCP line with a width of 500 nm at different annealing times, showing a periodic SiO*_x_* line structure under the optimum annealing condition (at 85°C for 60 min). Figure 5c and Figure S12 (Supporting Information) show the defect density of a self‐assembled 16‐nm‐SiO*_x_* line structure at various times. Figure 5d shows a wave‐shaped SD45 pattern consisting of 16‐nm‐SiO*_x_* lines under the optimum annealing conditions. Figure S13 (Supporting Information) shows an optimization process of annealing conditions for cylinder‐forming SD45 BCP, indicating the optimum annealing time (60 min) and temperature (85 °C). A cylinder‐forming SD45 BCPs require a longer annealing time at a higher temperature than sphere‐forming SD56 BCP. The reason is that BCP with a higher volume fraction commonly needs higher annealing temperature to obtain ordered nanostructures. In general, BCPs with smaller MW are used in the directed self‐assembly (DSA) process to obtain a narrower SiO*_x_* line structure. Figure 5e shows a templated SD28 BCP wave line pattern consisting of a sub‐10 nm SiO*_x_* line structure on a microscale wave pattern, showing a narrower line structure than the SD45 case. Figures S14 and S15 (Supporting Information) present other discrete microscale SD28 BCP patterns consisting of sub‐10 nm SiO*_x_* line structures, showing relatively well‐defined nano‐in‐micro patterns of line and dot‐in‐hole structure. Here, it should also be emphasized that various functional di‐BCPs and/or tri‐BCPs can be widely used as replication materials for the T‐nTP method to obtain periodic nano‐in‐micro patterns, depending on the desired circuit design of the pattern size and shape. Based on the results for the patterning of BCP by the T‐nTP method, to produce well‐organized nano‐in‐micro patterns on an eight‐inch substrate effectively, we used a laminating system capable of providing both uniform pressure and heat. Figure 5f shows the results of the microscale patternization of ultra‐thin BCP nanopatterns via the T‐nTP process on an eight‐inch substrate when using the heat‐rolling press machine. After the replication of the self‐assembled BCP thin film on the eight‐inch Si mold with the help of adhesive PI film, the replicated eight‐inch SD45 BCP thin film was successfully transfer‐printed onto a flexible and transparent PET surface using a laminating system. However, at an eight‐inch wafer scale, defects were often observed at the center and edge sides of the transfer‐printed BCP patterns, as shown in Figure S16 (Supporting Information) The irregular pattern structures may be due to non‐uniform BCP film thickness and/or low solvent injection rate in the annealing process of BCP before the transfer‐printing process. To obtain ordered BCP patterns on an eight‐inch wafer scale, it is necessary to conduct further BCP studies about DSA process on the eight‐inch Si mold. In addition, to obtain multi‐layered nanostructures, we employed a micro‐patterned Si mold with a greater depth (≈250 nm). Well‐defined multi‐layered BCP film was formed on the PET surface by an attaching and detaching process with the laminator, as shown in Figure 5g. Figure S17 (Supporting Information) shows the sequence of the processing steps used during the pattern‐transfer‐printing of multi‐layered BCP film on the eight‐inch wafer scale. This T‐nTP process used with BCP thin films is highly productive because the micro‐patterned eight‐inch Si mold is permanently reusable, allowing the BCP film to be repeatedly replicated through the replication process.

## 3 Conclusion

In summary, we demonstrated a very useful dual nanopatterning method that effectively generates well‐ordered sub‐20 nm structures in micro‐patterns on the eight‐inch wafer scale by the transfer‐printing of patterned ultra‐thin BCP film onto various substrates. We successfully achieved individual pattern formation of self‐assembled SiO*_x_* dot and line structures with good functionalities through a replication process by employing a combined patterning method of T‐nTP and DSA of Si‐containing BCPs. We also showed various complex pattern geometries of nano‐in‐micro structures over a large patterning area, specifically angular lines, wave lines, rings, dot‐in‐hole patterns, and dot‐in‐honeycomb patterns consisting of periodic sub‐20 nm SiO*_x_* structures. We expect that this state‐of‐the‐art dual nanopatterning process combined with other emerging nanopatterning methods will be widely used for the high‐throughput and high‐resolution nanofabrication of various electronic devices with complex circuits.

## 4 Experimental Section

### Fabrication of a Micro‐Patterned Si Master Template

The Si master template with depths of 40 and 250 nm for the BCP self‐assembly was fabricated using a conventional KrF photolithography process followed by reactive ion etching (RIE). A positive photoresist (PR, Dongjin Semichem Co. Ltd.) with 400 nm‐thickness was spin‐casted on an eight‐inch Si wafer. The PR film on the wafer was then exposed using a KrF scanner (Nikon, NSR‐S203B) and developed using a developer solution (tetramethylammonium hydroxide, Dongjin Semichem Co. Ltd.). The remaining PR patterns were used as an etch mask to pattern the Si surface with CF_4_ by means of RIE (working pressure: 9 mTorr, plasma power: 180 W).

### BCP Self‐Assembly within the Micro‐Patterned Si Guiding Template

Prior to the BCP self‐assembly process, the surface of the Si substrate with micro‐patterned complex shapes was treated by hydroxyl‐terminated PDMS with a molecular weight of 5 kg mol^−1^ at 150 °C for 2 h in a vacuum oven. A PDMS‐brush treatment was employed to separate the BCP films easily from the Si master template. BCPs (SD56, SD45, and SD28) with (corresponding) PDMS volume fractions (*f*_PDMS_) of 16%, 33.7%, and 32.1% were used. To obtain mono‐layered SiO*_x_* nanostructures, the BCPs were dissolved in a toluene solvent, yielding 0.9 wt.% (SD56) and 1.0 wt.% (SD45 and SD28), respectively. All polymers (BCPs and hydroxyl‐terminated PDMS) were purchased from Polymer Source Inc. in Canada. The BCP solutions were spin‐coated onto Si guiding templates. BCP ultra‐thin films with a thickness of ≈40 nm were annealed in a toluene vapor atmosphere under the optimum conditions (SD56: 65 °C for 10 min, SD28 and SD45: 85 °C for 60 min) to obtain the micro‐separated desired BCP morphologies.

### Direct Patterning of Self‐Assembled Ultrathin BCP Film

A solvent‐annealed ultra‐thin BCP layer was separated from the Si master template using an adhesive PI film (3 M Inc.). To transfer the BCP film onto target substrate, a heat‐rolling press system (LAMIART‐470 LSI, GMP Corp.) capable of injecting both uniform heat and constant pressure was used. This system has four rolls encapsulated by elastic silicone rubber, and the rolling speed is controllable from 200 to 1,500 mm min^−1^. The self‐assembled BCP layer could be transferred onto the desired substrate by passing it between the heated rolls. The printing speed in this case was set to 330 mm min^−1^. After the patterning process, the transfer‐printed ultra‐thin BCP film was etched by CF_4_ plasma (gas flow rate = 30 sccm, working pressure = 15 mTorr, power = 60 W, and etching time = 25 s) followed by O_2_ plasma (gas flow rate = 30 sccm, working pressure = 15 mTorr, power = 60 W, and etching time = 30s) using an RIE system to remove the segregated top‐PDMS and the PS matrix, respectively, resulting in SiO*_x_* (oxidized PDMS) nanostructures with a sub‐20 nm resolution.

### Characterization

Various microscale pattern structures consisting of the self‐assembled sub‐20 nm ultrafine nanostructures were observed with a field emission scanning electron microscope (FE‐SEM, TESCAN MIRA3) at a high acceleration voltage (≈5–10 kV) and a working distance of 3–5 nm. The number of defects (isolated line, junction, and terminal point) were manually counted using an ImageJ software, according to the typical method as previously reported.^[60]^

## Conflict of Interest

The authors declare no conflict of interest.

## Supporting information

Supporting Information

## Data Availability

The data that support the findings of this study are available in the supplementary material of this article.
