# Supplementary material for: Direct Printing of Ultrathin Block Copolymer Film with Nano‐in‐Micro Pattern Structures
Source: Adv Sci (Weinh). 2023 Aug 21;10(29):2303412. doi: 10.1002/advs.202303412 (PMC10582423; doi:10.1002/advs.202303412)
Supplement: Supplementary file 1 — Supporting Information [file ADVS-10-2303412-s001.pdf]

ADVANCED SCIENCE
Open Access

Supporting Information

for *Adv. Sci.*, DOI 10.1002/advs.202303412

Direct Printing of Ultrathin Block Copolymer Film with Nano-in-Micro Pattern Structures

Tae Wan Park, Young Lim Kang, Eun Bin Kang, Hyunsung Jung, Seoung-Ki Lee, Geon-Tae Hwang, Jung Woo Lee, Si-Young Choi, Sahn Nahm, Se-Hun Kwon, Kwang Ho kim* and Woon Ik Park**

Supporting Information

Direct Printing of Ultrathin Block Copolymer Film with Nano-in-Micro Pattern Structures

Tae Wan Park[1,2†], Young Lim Kang[2†], Hyunsung Jung[3], Seoung-Ki Lee[4], Geon-Tae Hwang[2], Jung Woo Lee[4], Si-Young Choi[5], Sahn Nahm[1], Se-Hun Kwon[4], Kwang Ho kim[4,6]*, and Woon Ik Park[2]**

[†]These authors contributed equally to this work.

[1]Department of Materials Science and Engineering, Korea University, Seoul 02841, Republic of Korea
[2]Department of Materials Science and Engineering, Pukyong National University (PKNU), 45 Yongso-ro, Nam-gu, Busan 48513, Republic of Korea
[3]Nano Convergence Materials Center, Korea Institute of Ceramic Engineering & Technology (KICET), Jinju 52851, Republic of Korea
[4]School of Materials Science and Engineering, Pusan National University (PNU), Busan 46241, Republic of Korea
[5]Department of Materials Science and Engineering, Pohang University of Science and Technology (POSTECH), Pohang 37673, Republic of Korea
[6]Global Frontier R&D Center for Hybrid Interface Materials (HIM), Pusan National University, Busan 46241, Republic of Korea

Keywords: nanotransfer printing, nanopatterning, wafer scale, block copolymer, self-assembly

Table of contents (ToC)

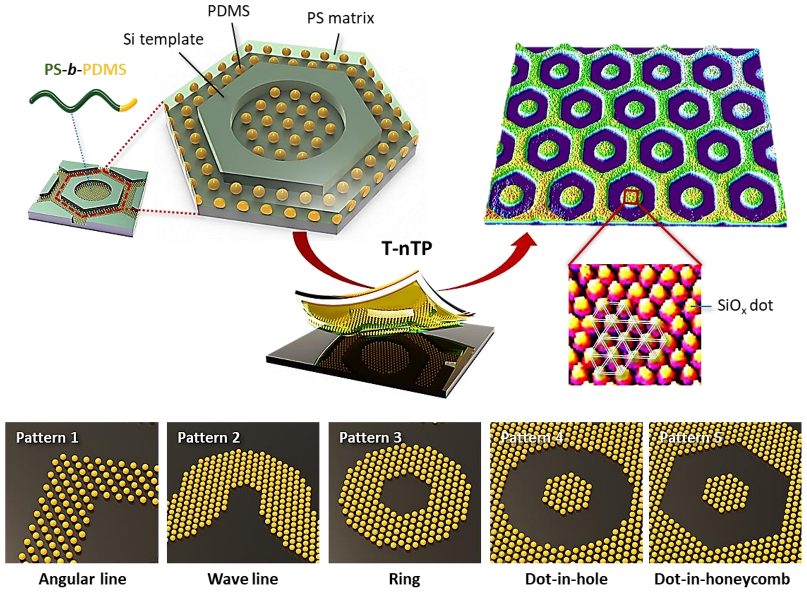

Figure S1. Sequential process for the fabrication of nano-in-micro pattern structures.

Figure S2. Transfer-printed ultra-thin BCP lines.

Figure S3. Self-assembled BCP on a planar surface.

Figure S4. Multiple BCP line patterns consisting of self-assembled SiO_x spheres.

Figure S5. Sequential cross-sectional SEM images for multi-layered BCP film at 2 wt% before dry-etching process.

Figure S6. Ultra-thin replica BCP layer on an adhesive PI film.

Figure S7. Temperature dependency on the transfer yield of ultra-thin BCP film.

Figure S8. Center-to-center size distribution of transfer-printed nut-shaped SiO_x nanodot patterns.

Figure S9. Si guiding templates with complex pattern geometries.

Figure S10. Unusual and complex nano-in-micro pattern structures by T-nTP of self-assembled BCP.

Figure S11. Self-assembled SiO_x line structure with a line width of sub-20-nm on a planar surface without a guiding template.

Figure S12. Dependency on the annealing time of the self-assembled SiO_x line structures.

Figure S13. Optimization process of annealing conditions for cylinder-forming SD45 BCP.

Figure S14. Transfer-printed SiO_x lines within the individual microscale line pattern when using a cylinder-forming SD28 BCP.

Figure S15. Transfer-printed ring-shaped SiO_x lines within the complex microscale dot-in-hole pattern when using a cylinder-forming SD28 BCP.

Figure S16. Defects on the center and edge sides of the transfer-printed BCP patterns at an eight-inch wafer scale.

Figure S17. Procedure for the pattern formation of multi-layered BCP film at an 8-inch wafer scale.

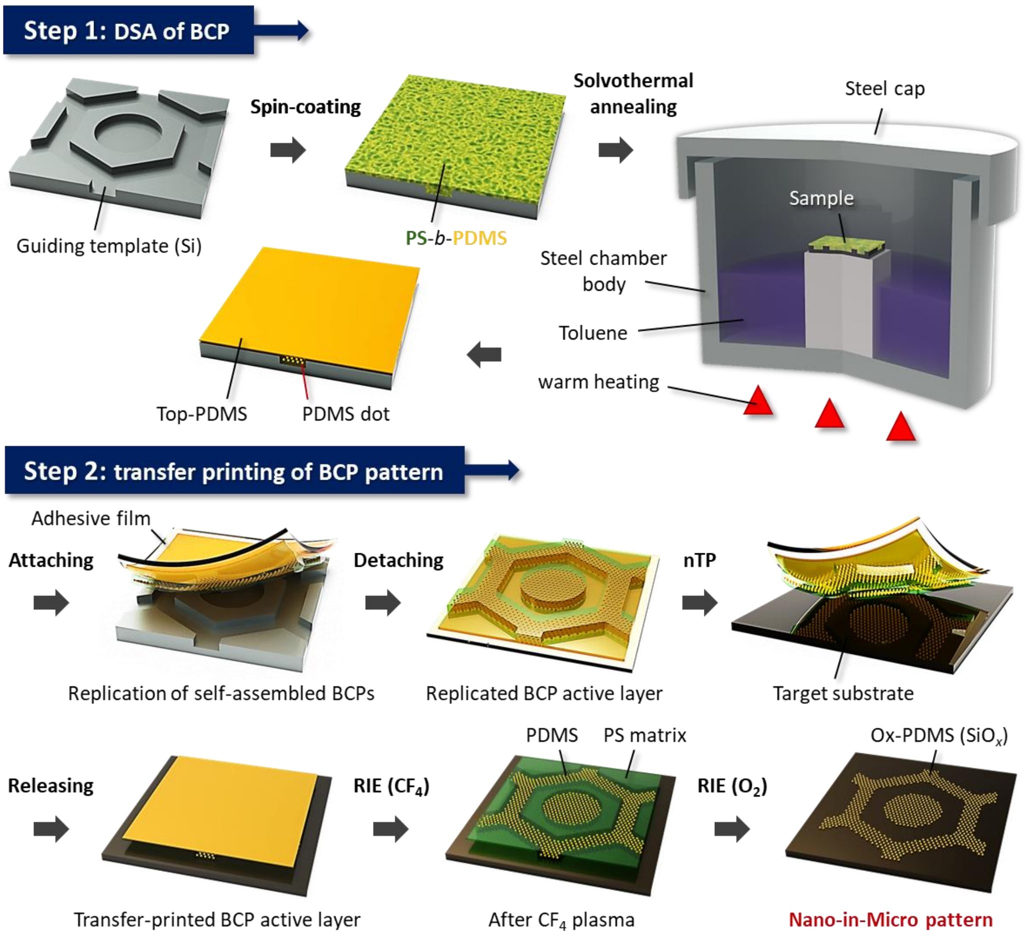

Figure S1. Sequential process for the fabrication of nano-in-micro pattern structures. (Step 1: DSA of BCP) The BCP solution is spin-coated onto a Si guiding template fabricated by the conventional photolithography process. The coated BCP thin film is solvothermal-annealed at a warm temperature (~ 65℃) using a stainless-steel chamber that can provide solvent vapor into the BCP film. (Step 2: transfer-printing of BCP patterns) A replica BCP pattern is generated by attaching and detaching using an adhesive PI film. The functional BCP pattern is printed onto the target substrate through the T-nTP process, after which transfer-printed replica BCP film is etched by CF_4 plasma followed by O_2 plasma. The oxidized PDMS nanodots-in-unusual micro pattern structure (nano-in-micro pattern) is successfully obtained.

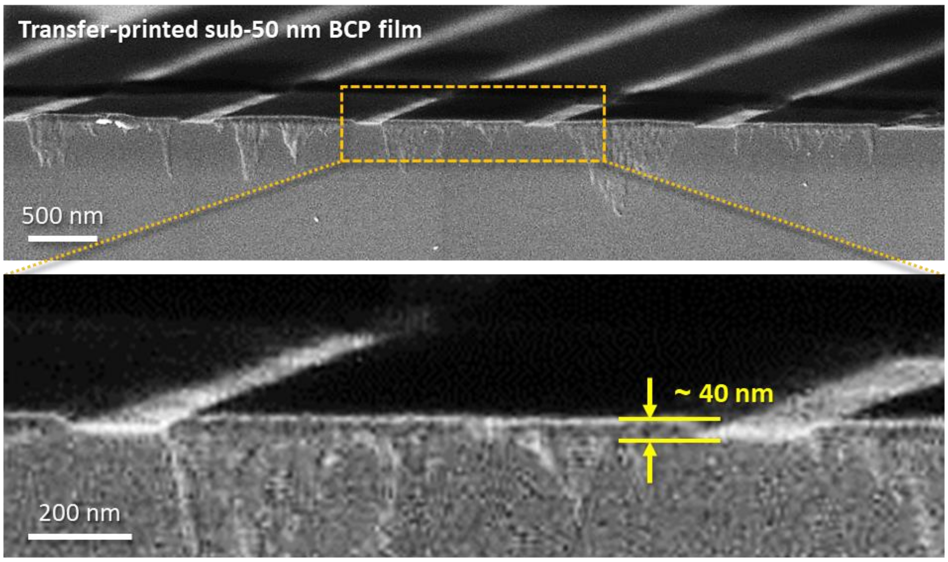

Figure S2. Transfer-printed ultra-thin BCP lines. SEM image of discrete BCP line patterns with a width of 1 µm.

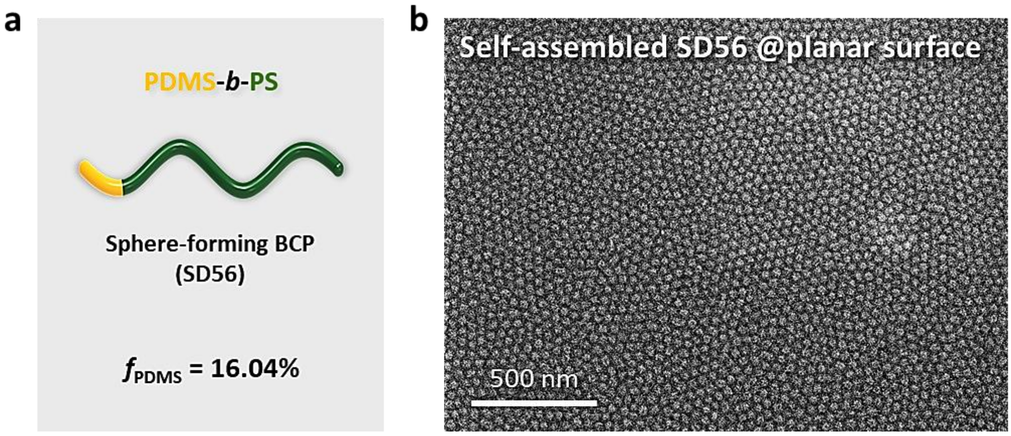

Figure S3. Self-assembled BCP on a planar surface. (a) Sphere-forming SD56 BCP. (b) SEM image of hexagonally arranged SiO_x dots.

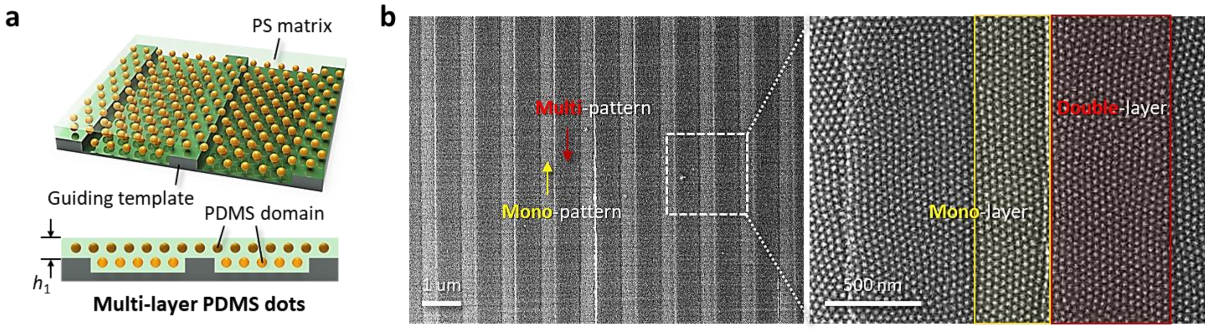

Figure S4. Multiple BCP line patterns consisting of self-assembled SiO_x spheres. (a) Schematic images of self-assembled multi-layer PDMS dots. (b) Transfer-printed multi-layer BCP patterns. Multi-layer microscale BCP line patterns can be obtained by controlling the BCP film thickness.

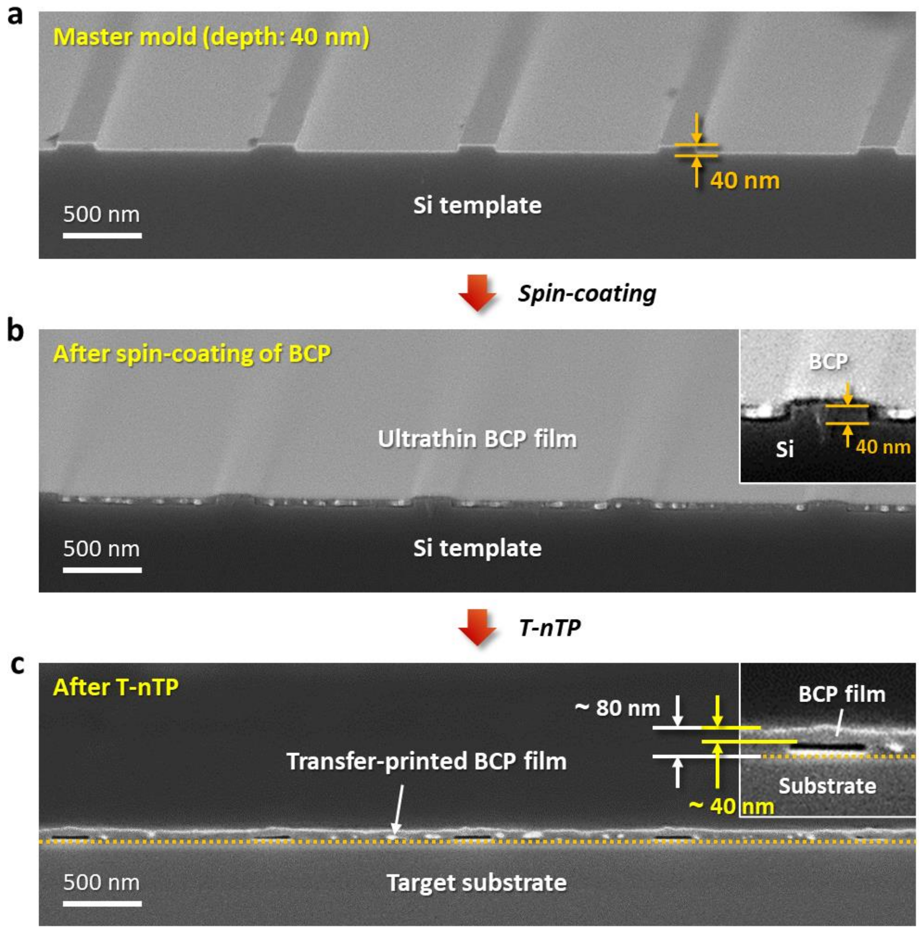

Figure S5. Sequential cross-sectional SEM images for multi-layered BCP film at 2 wt% before dry-etching process. (a) Si mold with a depth of 40 nm, (b) After spin coating of BCP, (c) After transfer-printing. When using the SD56 BCP solution with a higher weight percent, the transfer printing result shows an interconnected BCP film rather than individual BCP lines (thickness of transfer-printed BCP film: ~ 80 nm).

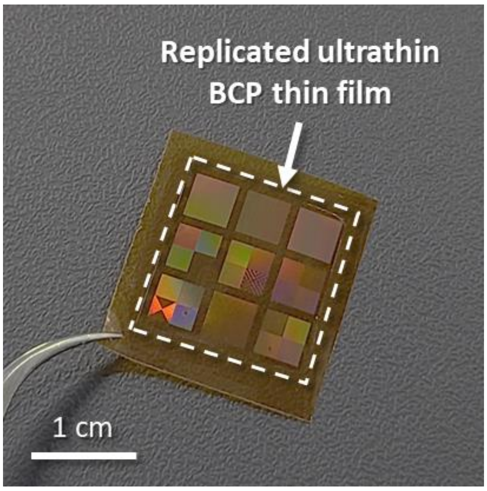

Figure S6. Ultra-thin replica BCP layer on an adhesive PI film. Photograph of the replicated BCP film from the micro-patterned Si mold.

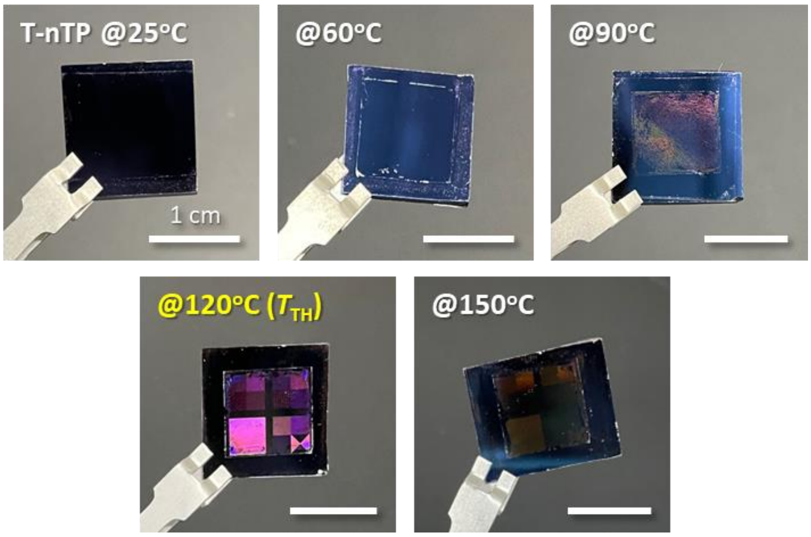

Figure S7. Temperature-dependency on the transfer yield of ultra-thin BCP film. Photographs of transfer-printed BCP films from 25℃ to 150℃. The threshold temperature for the successful patterning of the ultra-thin BCP film is 120℃.

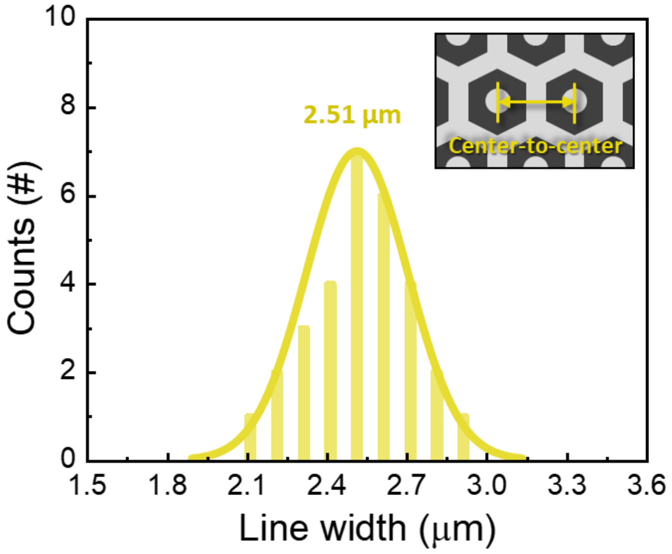

Figure S8. Center-to-center size distribution of transfer-printed nut-shaped SiO_x nanodot patterns. Mean value of center-to-center distance is 2.51 µm. The error range of the transfer-printed nano-in-micro patterns is less than 0.5%.

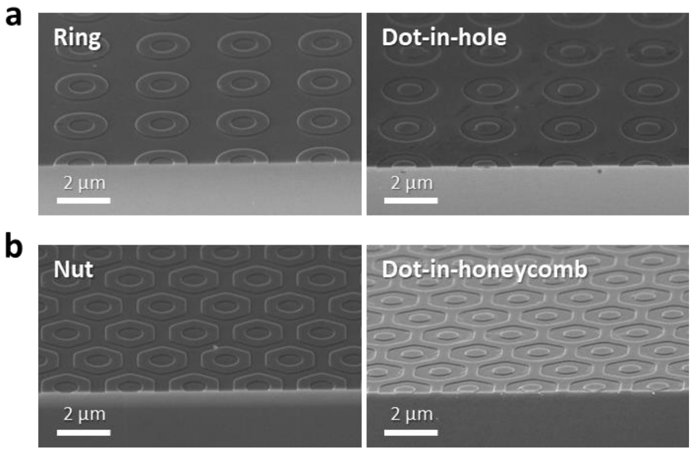

Figure S9. Si guiding templates with complex pattern geometries. (a) Surface-patterned ring and reverse-ring (dot-in-hole) templates. (b) Nut and reverse-nut (dot-in-honeycomb).

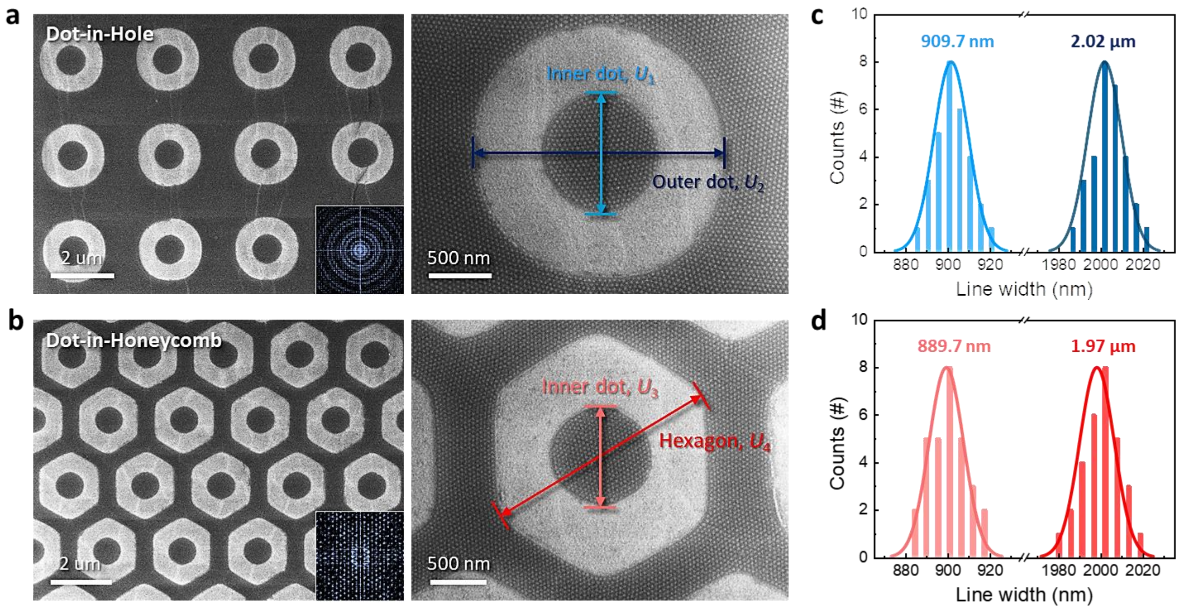

Figure S10. Unusual and complex nano-in-micro pattern structures by the T-nTP of self-assembled BCP. Transfer-printed microscale (a) dot-in-hole and (b) dot-in-honeycomb patterns composed of self-assembled SiO_x nanodots. (c & d) Uniformity of the transfer-printed inner units (dot) and outer units (dot and hexagon), showing mean size values of 901.7 nm (Unit 1, U_1), 2.02 µm (U_2), 889.7 nm (U_3), and 1.97 µm (U_4), respectively. The maximum error range of all printed units is less than 0.2%.

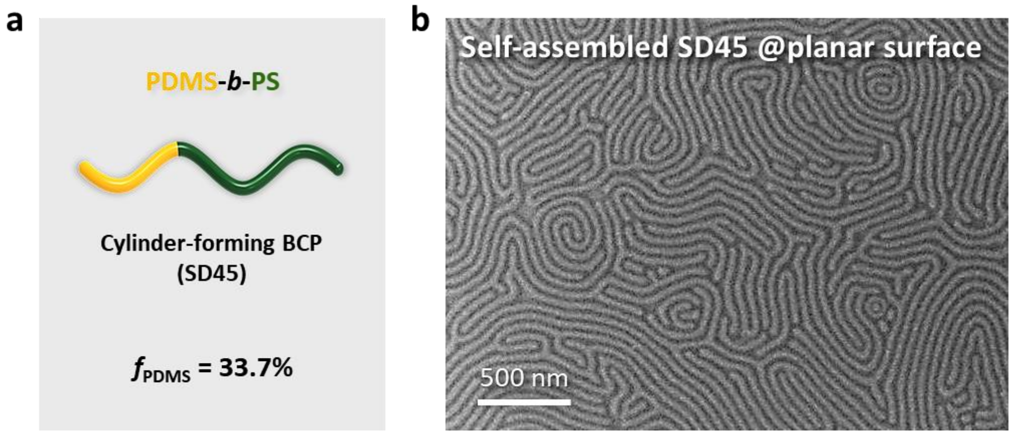

Figure S11. Self-assembled SiO_x line structure with a line width of sub-20-nm on a planar surface without a guiding template. (a) Schematic of cylinder-forming SD45 BCP. (b) SEM image of disordered SiO_x lines.

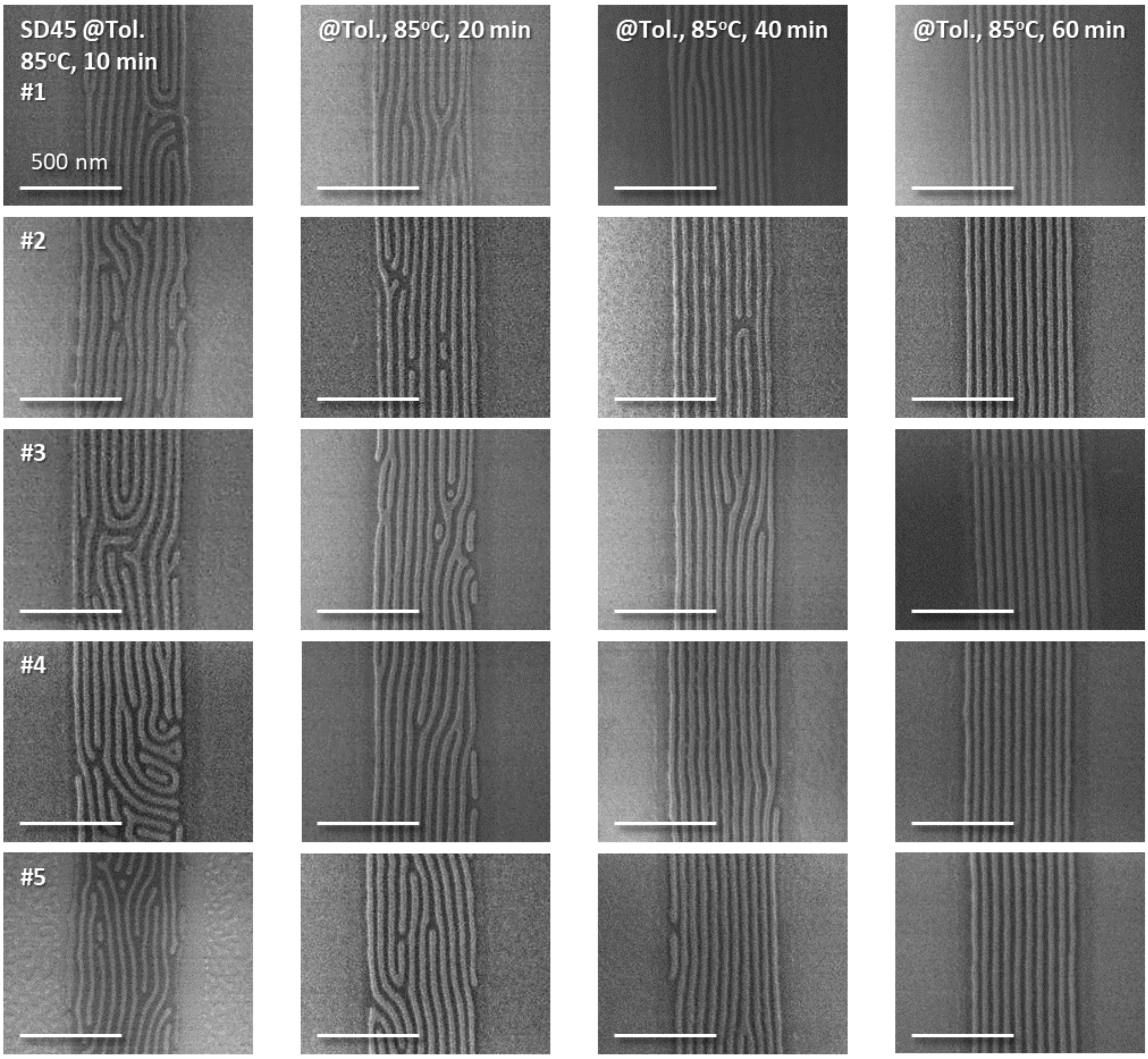

Figure S12. Dependency on the annealing time of the self-assembled SiO_x line structures. Periodic SD45 line structures were obtained at annealing time of 60 mins.

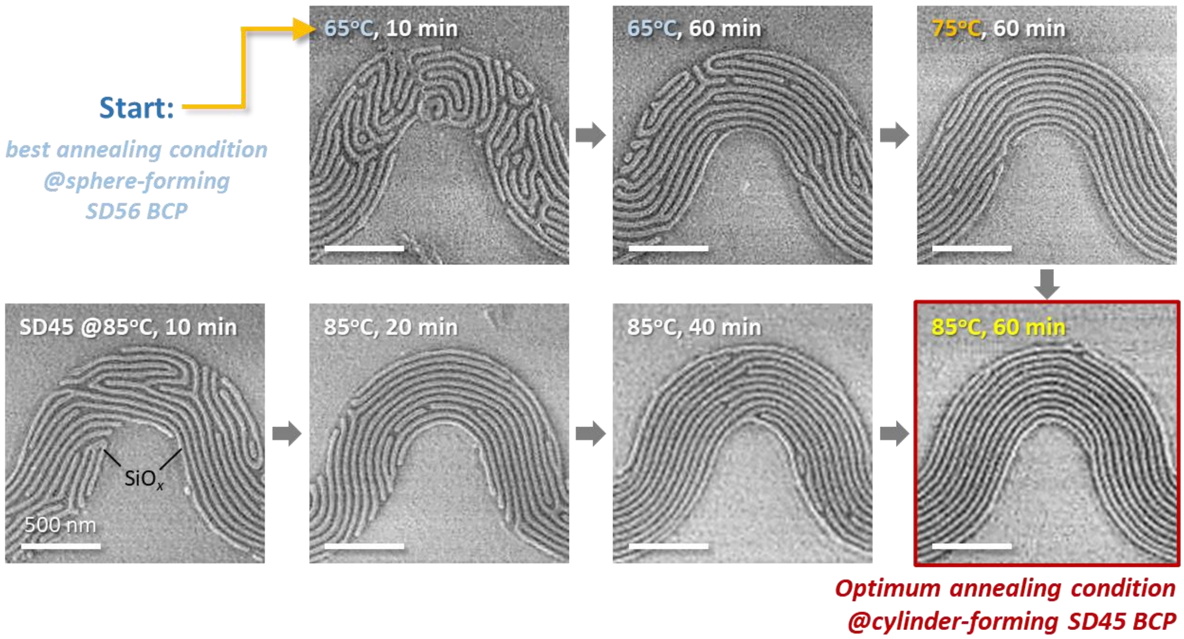

Figure S13. Optimization process of annealing conditions for cylinder-forming SD45 BCP. The self-assembled sub-20 nm SiO_x lines within a wave-shaped SD45 BCP pattern at varied annealing times, indicating the optimum annealing time (60 min) and temperature (85℃).

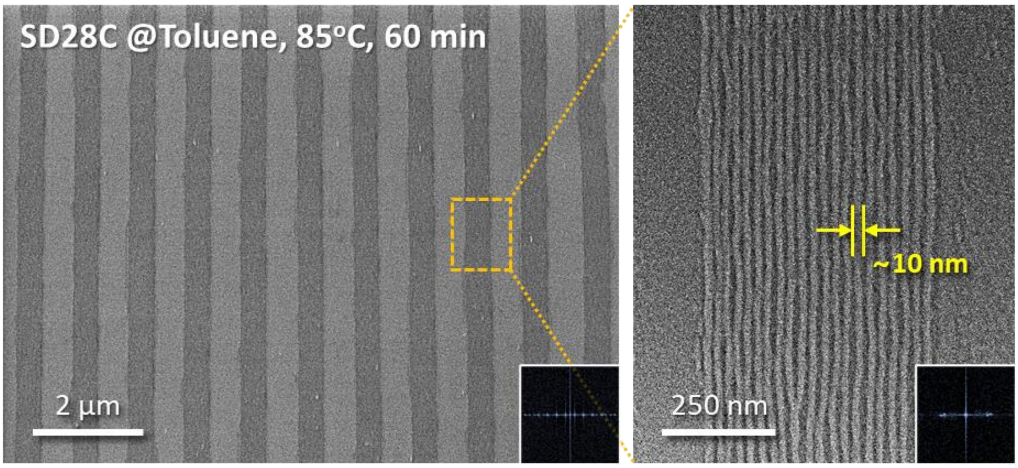

Figure S14. Transfer-printed SiO_x lines within the individual microscale line pattern when using a cylinder-forming SD28 BCP.

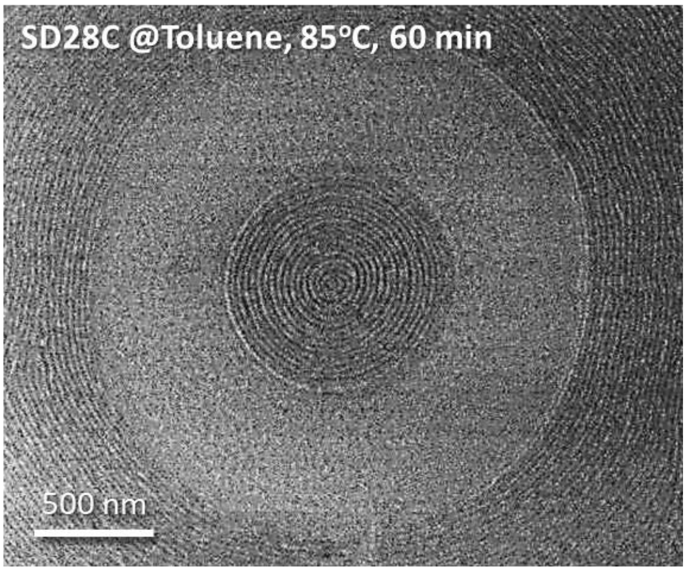

Figure S15. Transfer-printed ring-shaped SiO_x lines within the complex microscale dot-in-hole pattern when using a cylinder-forming SD28 BCP.

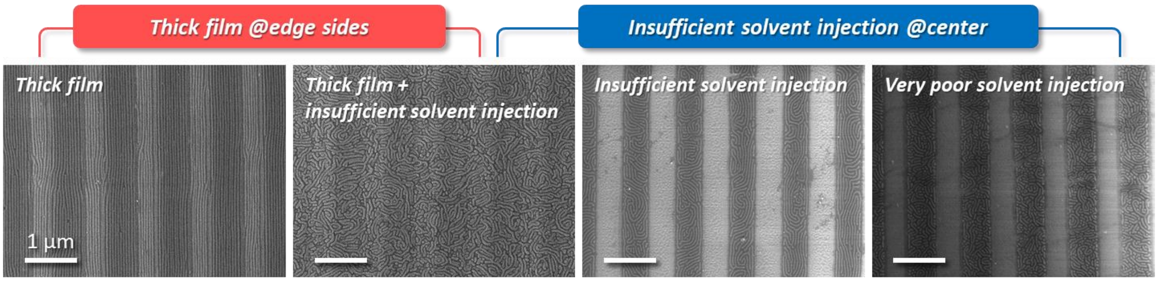

Figure S16. Defects on the center and edge sides of the transfer-printed BCP patterns at an eight-inch wafer scale. The irregular pattern structures may be due to non-uniform BCP film thickness and/or low solvent injection rate in the annealing process of BCP before transfer-printing process.

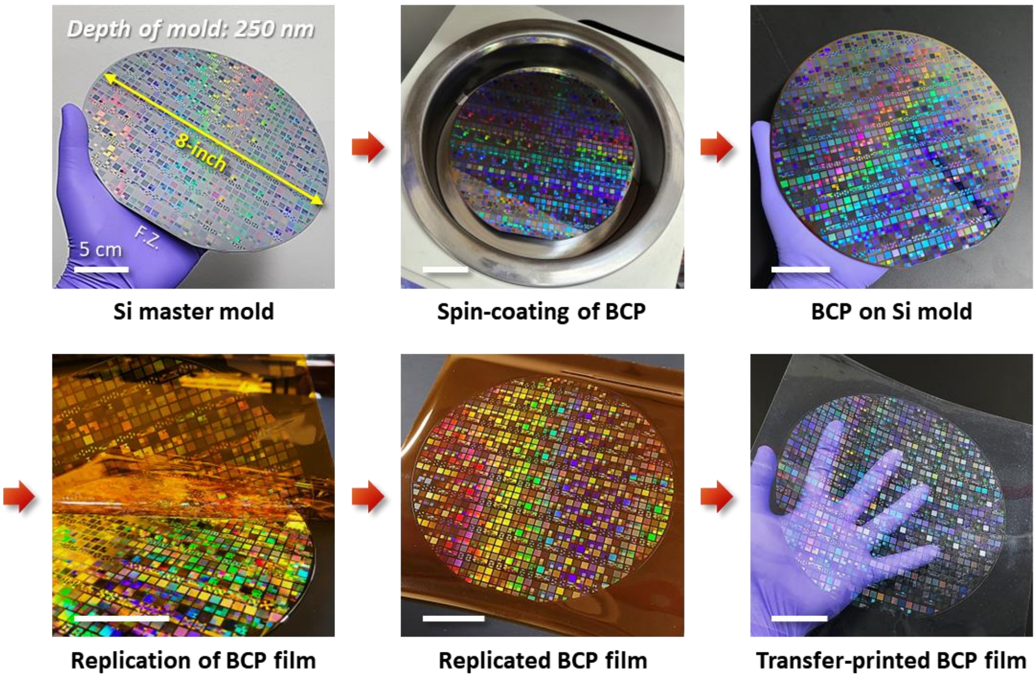

Figure S17. Procedure for the pattern formation of multi-layered BCP film on the eight-inch wafer scale. The depth of micro-patterned Si mold is ~ 250 nm.
